# Tetrahydroxystilbene Glucoside Effectively Prevents Apoptosis Induced Hair Loss

**DOI:** 10.1155/2018/1380146

**Published:** 2018-04-02

**Authors:** Lulu Chen, Huichuan Duan, Feng Xie, Zhen Gao, Xiaoli Wu, Fanfan Chen, Wei Wu

**Affiliations:** ^1^Department No. 16, Plastic Surgery Hospital, Chinese Academy of Medical Sciences and Peking Union Medical College, Beijing, China; ^2^Department of Plastic and Reconstructive Surgery, Shanghai 9th People's Hospital, Shanghai Jiao Tong University School of Medicine, Shanghai, China

## Abstract

The effect of* Polygonum multiflorum* against hair loss has been widely recognized. 2,3,5,4′-Tetrahydroxystilbene-2-O-*β*-D-glucoside (TSG) is the main component of* Polygonum multiflorum*; however, its role in hair regeneration has not been established. To evaluate the hair growth-promoting activity of TSG, depilated C57BL/6J mice were topically treated with normal saline, TSG, Pifithrin-*α*, Minoxidil for 2 weeks. In this study, we identified that p53, Caspase-3, Active Caspase-3, and Caspase-9 were obviously upregulated in the skin of human and mice with hair loss by western blot analysis. Depilated mice treated with TSG showed markedly hair regrowth. TUNEL^+^ cells were also reduced in mice with TSG. These changes were accompanied with inhibition of Fas, p53, Bax, Active Caspase-3, and Procaspase-9 activities. These results demonstrated that TSG exerts great hair regrowth effect on hair loss, which was probably mediated by inhibition of p53, Fas, and Bax induced apoptosis.

## 1. Introduction

Hair follicle is a complete cutaneous miniorgan that shows intriguing features of cyclic activity in postnatal life. It is clear that, after a prolonged period of growth (anagen), hair follicle spontaneously turns into a phase of apoptosis-driven involution (catagen) until the follicle reundergoes anagen via a resting phase (telogen) [1]. Premature differentiation of keratinocytes, along with the activation of a variety of apoptotic signaling pathways, is a characteristic of hair follicle catagen [2–4]. Hair loss is the result of recurrent premature termination of follicle growth and repeated induction of apoptosis-regression in catagen [5, 6].

At present, clinical treatments for hair loss can be classified into two kinds: surgical and medical therapies. Although hair transplantation surgery has reliable effects, there are still some shortages that limit its widespread application including invasive surgical process, high price, and limited source of donor follicles [7]. Up to now, only finasteride and Minoxidil have been approved by the FDA [8]. However, the application of these two drugs is limited owing to their limited and transient effects, with unexpected side effects and high recurrence rate [9, 10]. Given their limitation, it is needed to develop a better more effective material to prevent hair loss.

*Polygonum multiflorum* is a well-known Chinese herbal medicine which has been traditionally used to treat hair loss and hair blackening [11]. Some studies have found that hair growth promotion effects of* Polygonum multiflorum* were probably mediated by several cytokines, such as Sonic Hedgehog (SHH), insulin-like growth factor-1 (IGF-1), hepatocyte growth factor (HGF), fibroblast growth factor-7 (FGF-7), and *β*-catenin [11, 12]. Besides, Han et al. have shown that marked beneficial effects of* Polygonum multiflorum* on hair quality and appearance were through upregulating *α*-melanocyte stimulating hormone (*α*-MSH), melanocortin 1 receptor (0MC1R), and tyrosinase (TYR) [13].

However, it is still not known which compound of* Polygonum multiflorum* contributes to promoting hair growth, nor has any report talks about the exactly mechanism of the* Polygonum multiflorum* extract in treating hair loss. 2,3,5,4′-Tetrahydroxystilbene-2-O-*β*-D-glucoside (TSG) is the major active chemical constituent of the* Polygonum multiflorum* and has been proven to exhibit strong antioxidant, free radical scavenging activities, anti-inflammation, antiaging, neuroprotection, and antiapoptosis properties [14–17]. Previous study reported that TSG had significantly induced melanogenesis through elevating the level of p38 mitogen-activated protein kinase (MAPK) phosphorylation, which suggested that it might be an effective treatment for hair graying wherein hair graying was due to loss of melanization signals [14]. It is also reported that TSG increased the proliferation of dermal papilla cells in vitro [18]. Although TSG was reported to have various protective effects under many pathophysiological conditions, whether it can play an important role in improving hair loss has not been clearly clarified.

Here, hair growth-promoting activity of TSG was investigated in C57BL/6J mice and its possible molecular mechanism was also discussed in this study.

## 2. Materials and Methods

### 2.1. Human Samples

Human scalp samples were kindly donated from Shanghai 9th People's Hospital and all patients gave informed consent. Skin was from occipital (hairy) and frontal (bald) scalps of 10 age-matched people. All patients without scalp lesions were not receiving any topical or systemic therapy for hair loss within a year. The study was approved by the Ethics Committee of Shanghai 9th People's Hospital affiliated to Shanghai Jiao Tong University School of Medicine.

### 2.2. Animals and Treatments

Thirty-six-week-old C57BL/6J mice (Shanghai SLAC Laboratory Animal Co., Ltd., Shanghai, China) were randomly assigned to 5 groups (*n* = 6): Normal group, normal mice without any treatment; Vehicle group, depilated mice treated with normal saline; TSG group, depilated mice treated with 200 *μ*M TSG (National Institute for the Control of Pharmaceutical and Biological Products, China); p53 inhibitor group, which received 200 mM Pifithrin-*α* (Selleck Chemicals, USA); Minoxidil group, which was treated with 2% Minoxidil (Sigma, USA). All medicines were performed topically on the upper back once per day for 2 weeks. Afterwards, mice were sacrificed and dorsal skins were fixed in 4% paraformaldehyde (PFA) (Sigma) or frozen in liquid nitrogen for further study. All animal experiment protocols were approved by the Animal Experiment and Care Committee of Shanghai Jiao Tong University School of Medicine.

Five groups of mice were all treated with the same depilated model, which was induced as previously described [19]. Briefly, a melted wax/rosin mixture (1 : 1) under general anesthesia was used on the dorsal skin which could peel off all hair shafts and immediately induce all follicles to turn into homogeneous growth phase [19].

### 2.3. Hair Length, Hair Cover Skin Ratio, and Hair Follicle Numbers

Dorsal hairs were collected randomly at 2 weeks and 10 hairs of each mouse were randomly chosen to measure the average hair length of each group. At day 0, we took pictures of mice dorsal depilated skin. Two weeks later, hair growth areas were measured in each mouse to calculate hair cover skin ratio using Image-Pro Plus 6.0 software (Media Cybernetics, USA). Slides stained with H&E were imaged using a microscope (Leica, Germany). Six photos of 100x magnification were randomly chosen to calculate the hair follicle numbers.

### 2.4. Detection of Apoptotic Cells

Human scalp samples and mice dorsal skin were embedded in optimal cutting temperature compound (OCT), and 10 *μ*m thick cryosections were obtained using a Thermocryotome (Thermo, USA) and mounted on coated slides (Dako, Denmark). After washing OCT with phosphate buffered saline (PBS), terminal deoxynucleotidyl transferase dUTP nick end labeling (TUNEL) kit (Roche, Germany) was used to detect apoptosis cells according to the manufacturer's instructions. Images were obtained using a fluorescence microscope (Olympus, Japan).

### 2.5. Western Blot Analysis

Total protein lysates of skin samples were prepared with T-PER tissue protein extraction reagent (Pierce, USA). The prepared protein was subjected to SDS-polyacrylamide gel electrophoresis and subsequently transferred onto PVDF membranes (Bio-Rad, USA). The membranes were incubated with primary antibodies including anti-Bcl-2 (1 : 500), anti-Bax (1 : 500), anti-*β*-actin (1 : 1000), anti-p53 (1 : 500), anti-Caspase-3 (1 : 500), anti-Active Caspase-3 (1 : 500), anti-Caspase-9 (1 : 500), anti-Procaspase-9 (1 : 500), followed by incubation with appropriate HRP-conjugated secondary antibodies (Jackson ImmunoResearch, USA). All primary antibodies were purchased from Cell Signaling Technology. The protein bands were eventually visualized using an enhanced chemiluminescence (ECL) detection reagent (Amersham Pharmacia, USA).

### 2.6. Real-Time RT-PCR Analysis

Skin samples were lysed with 1 ml TRIzol (Invitrogen) and then transferred to 1.5 ml Eppendorf tubes. After adding 400 *μ*l chloroform, the solution was vigorously mixed for 30 s, placed on the ice for 15 min, and centrifuged at 12,000 rpm for 15 min at 4°C. The upper layer (clear, colorless) was mixed thoroughly with an equal volume of isopropyl alcohol. After centrifugation at 12,000 rpm for 10 min at 4°C, the supernatant was removed and the sediment was washed with 75% alcohol before centrifugation at 7,500 rpm for 15 min at 4°C. The concentration of RNA dissolved in 40 *μ*l DEPC-treated water was measured by spectrophotometry. The purified RNA sample (2 *μ*g) was reverse transcribed to cDNA using a reaction system (TaKaRa) containing 4 *μ*l Buffer, 1 *μ*l dNTPs, 1 *μ*l Oligo, 1 *μ*l reverse transcriptase, 1 *μ*l enzyme inhibitor, 12 *μ*l RNA, and H_2_O. The PCR amplification conditions were as follows: 30°C for 10 min followed by 42°C for 60 min; the completed reaction was maintained at 4°C.

Real-time RT-PCR analysis was performed in reaction system (10 *μ*l) consisting of 1 *μ*l upstream primers, 1 *μ*l downstream primers, 1 *μ*l cDNA, and 7 *μ*l DEPC-treated water. The following primers were used: Fas (5′- AGA AAT TCA GCC CGT TGG AGT-3′ and 5′- GTT GCA TCC ACC CAA ATC ACC-3′); Bax (5′-AGG ATG CGT CCA CCA AGA AG-3′ and 5′-CTT GGA TCC AGA CAA GCA GC-3′); Bcl-2 (5′-CTG AGT ACC TGA ACC GGC AT-3′ and 5′-CTG AGT ACC TGA ACC GGC AT-3′); Caspase-9 (5′-CAC CTT CCC AGG TTG CCA ATG-3′ and 5′-CAA GCC ATG AGA GCT TCG GA-3′); Caspase-3 (5′-GAG CTT GGA ACG GTA CGC TAA-3′ and 5′-GAG TCC ACT GAC TTG CTC CC-3′); p53 (5′-CAT CCT GGC TGT AGG TAG CG-3′ and 5′-CAT CCT GGC TGT AGG TAG CG-3′); and *β*-actin (5′- GTA CCA CCA TGT ACC CAG GC-3′ and 5′- AAC GCA GCT CAG TAA CAG TCC-3′). The reaction conditions were as follows: 95°C for 10 min, followed by 40 cycles of 95°C for 30 s, 60°C for 30 s, and 72°C for 45 s. Reactions were performed using the StrataGene Mx3000p (Agilent Technologies, USA).

### 2.7. HE Staining

Skin samples were fixed with 4% PFA and embedded in paraffin wax. The skin were then cut by microtome into 5 *μ*m thick slides and stained with haematoxylin and eosin (H&E).

### 2.8. Statistical Analysis

Data were shown as means ± standard deviations (SD). For quantitative comparison and analysis, values were performed utilizing paired *t*-tests between two groups and one-way analysis of variance (ANOVA) among three or more than three groups using SPSS version 19.0 (Chicago, IL, USA). *P* < 0.05 was considered statistically significant.

## 3. Results

### 3.1. Promotion Effects of TSG on the Recovery of Hair Loss

**Figure 1:**
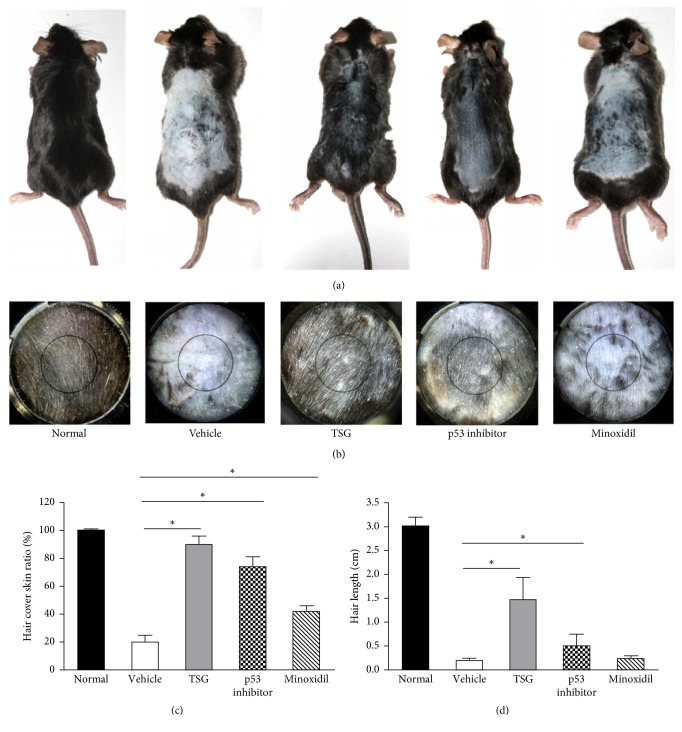
Morphological findings on the back of mice. Mice were treated with normal saline, TSG, Pifithrin-*α*, and Minoxidil for 2 weeks. ((a), (b)) morphological observation of new hair. Hair cover skin ratio (c) and hair length (d) were evaluated among five groups. ^*∗*^*P* < 0.01.

**Figure 2:**
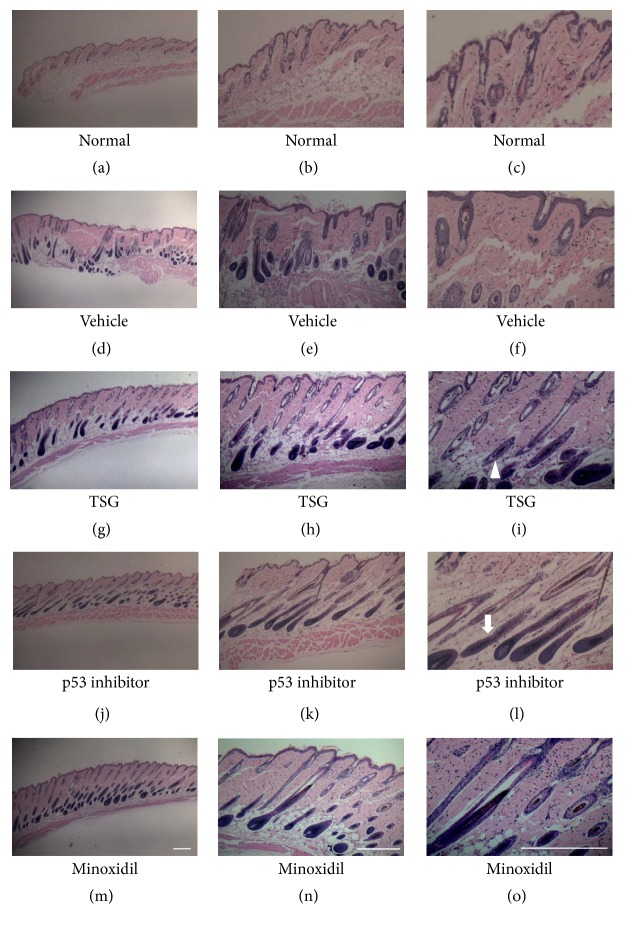
Histological changes of hair follicles after 2 weeks' treatment. Arrows: hair shaft. Arrowheads: hair follicle containing hair fiber. Bar = 250 *μ*m.

After 2 weeks, mice topically treated with TSG showed markedly hair regrowth, which showed near-complete recovery to normal levels. The depilated back skin was covered with black fur. In contrast, in vehicle and Minoxidil group, most of depilated skin remained hairless (Figures 1(a) and 1(b)). Furthermore, both TSG and p53 inhibitor group showed black skin color, while Minoxidil and vehicle group mice still contained gray skin color (Figures 1(a) and 1(b)). From hair covered skin areas and hair length observed, TSG treated mice owned 91%  ± 5.3% hair covered skin ratio and 1.40  ± 0.6 mm hair length, which showed the best promotion effects than other groups (Figures 1(c) and 1(d)). In the vehicle group, hair follicles were dystrophic and miniaturized (Figures 2(d), 2(e) and 2(f)). Treatment with TSG, p53 inhibitor, and Minoxidil increased the sizes and numbers of hair follicles and hair fibers (Figures 2(g)–2(o)) (Figure 4(a)).

### 3.2. Reduction of TUNEL^+^ Cells in Mice Treated with TSG

**Figure 3:**
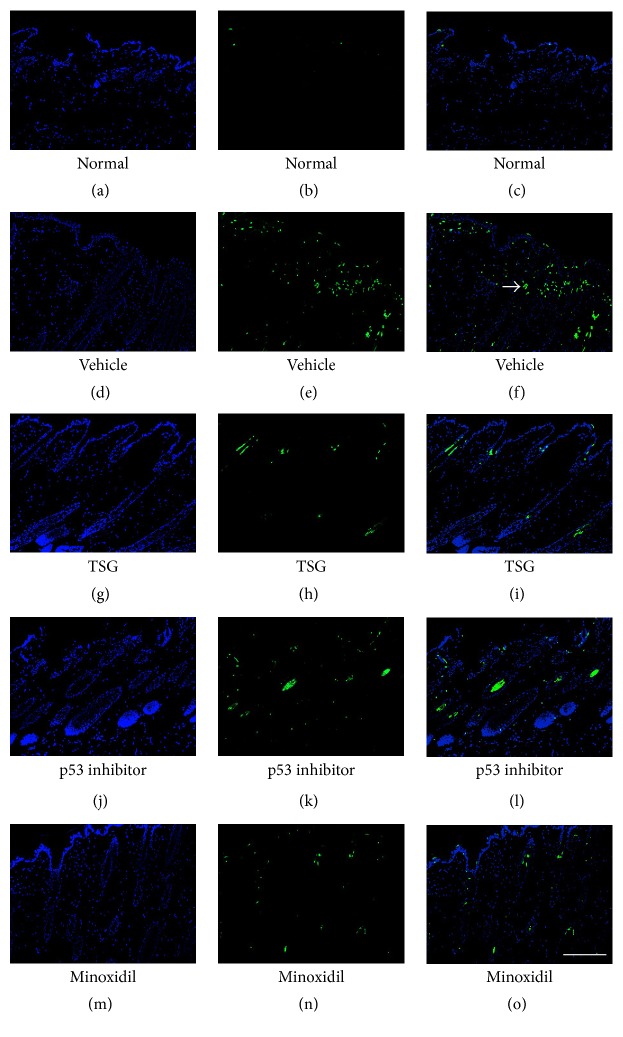
Expression of TUNEL^+^ cells after 2 weeks treatment. Arrow: TUNEL^+^ cells. Bar = 250 *μ*m.

**Figure 4:**
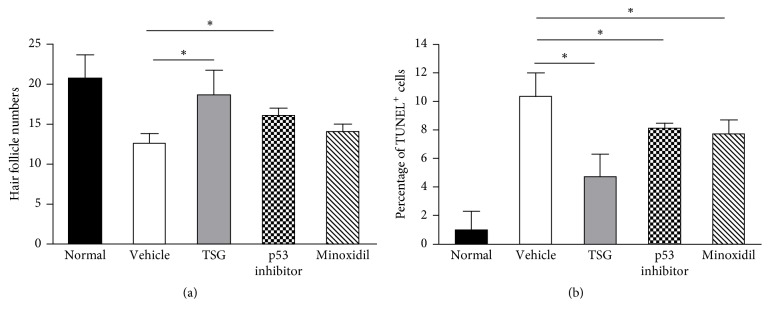
Comparison of hair follicle numbers and percentage of TUNEL^+^ cells among five groups. ^*∗*^*P* < 0.01.

TUNEL staining was performed to detect variation trends of apoptosis in five groups. In normal skin, only very few TUNEL^+^ cells were observed (Figures 3(a)–3(c)). On the contrary, TUNEL^+^ cells were obviously increased in mice of vehicle group (Figures 3(d)–3(f)). Meanwhile, depilated mice treated with TSG showed the least TUNEL^+^ cells when compared with vehicle, p53 inhibitor, and Minoxidil group (Figure 4(b)). These findings revealed that TSG showed more powerful anti-apoptotic activity than p53 inhibitor and minoxidil.

### 3.3. Molecular Mechanism of TSG on Inhibition of Apoptosis

**Figure 5:**
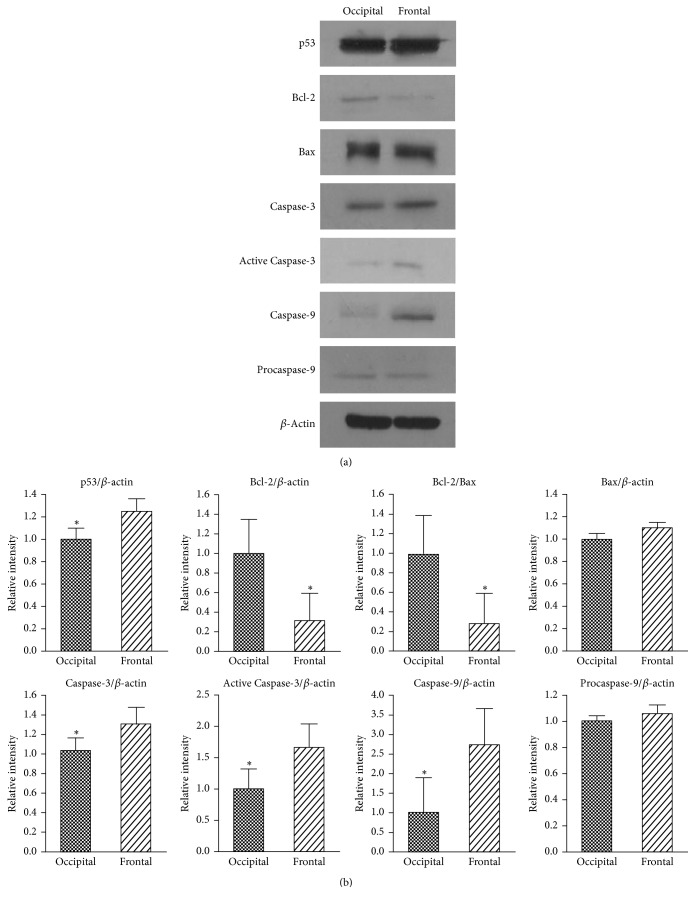
Expression of apoptosis related proteins in human frontal and occipital scalps by western blot. p53, Caspase-3, Active Caspase-3, and Caspase-9 were activated and Bcl-2 was suppressed in frontal scalp. ^*∗*^*P* < 0.05.

**Figure 6:**
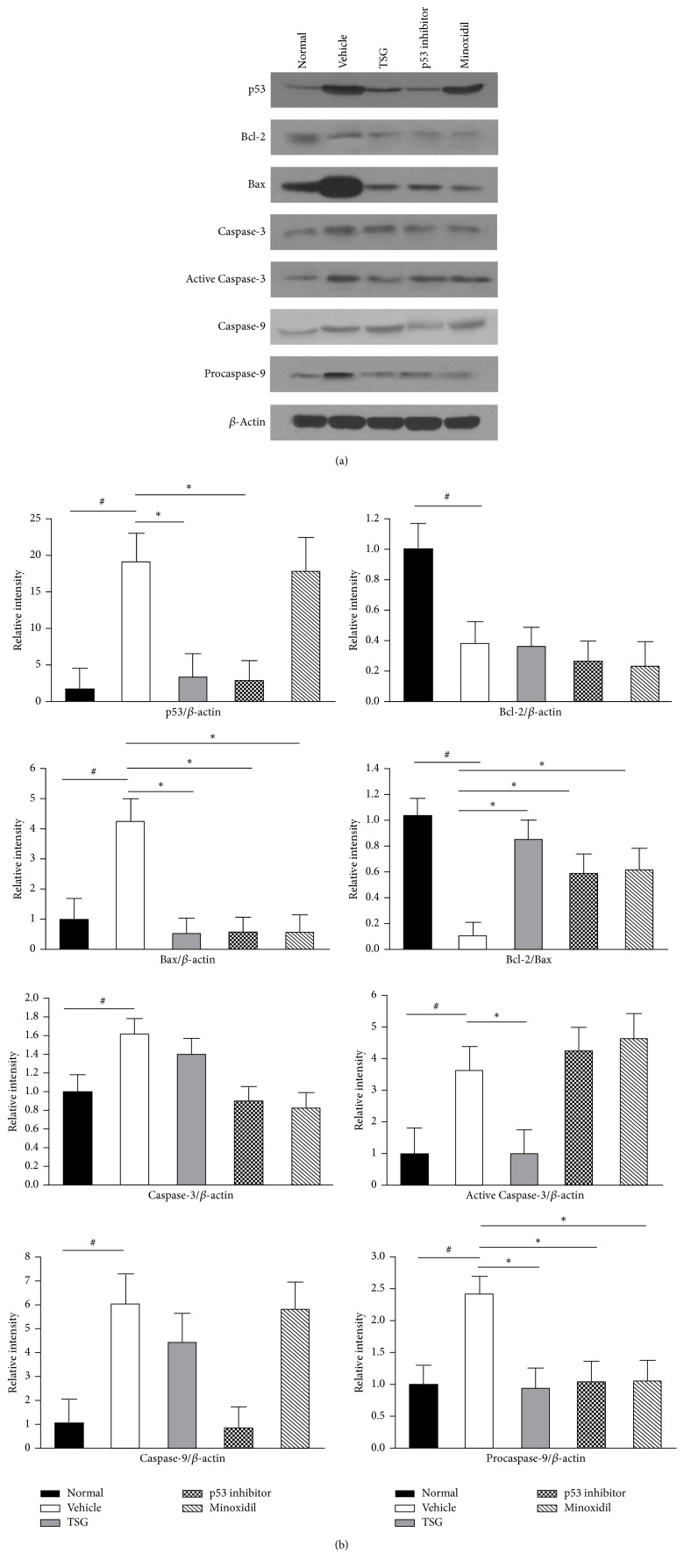
Expression of apoptosis related proteins in five groups of mice by western blot. p53, Bax, Caspase-3, Active Caspase-3 and Caspase-9, and Procaspase-9 were upregulated and Bcl-2 was reduced in depilated mice treated with normal saline compared with normal mice. Expression levels of p53, Bax, Active Caspase-3, and Procaspase-9 were decreased after TSG treatment. ^*∗*^*P* < 0.01. ^#^*P* < 0.01.

**Figure 7:**
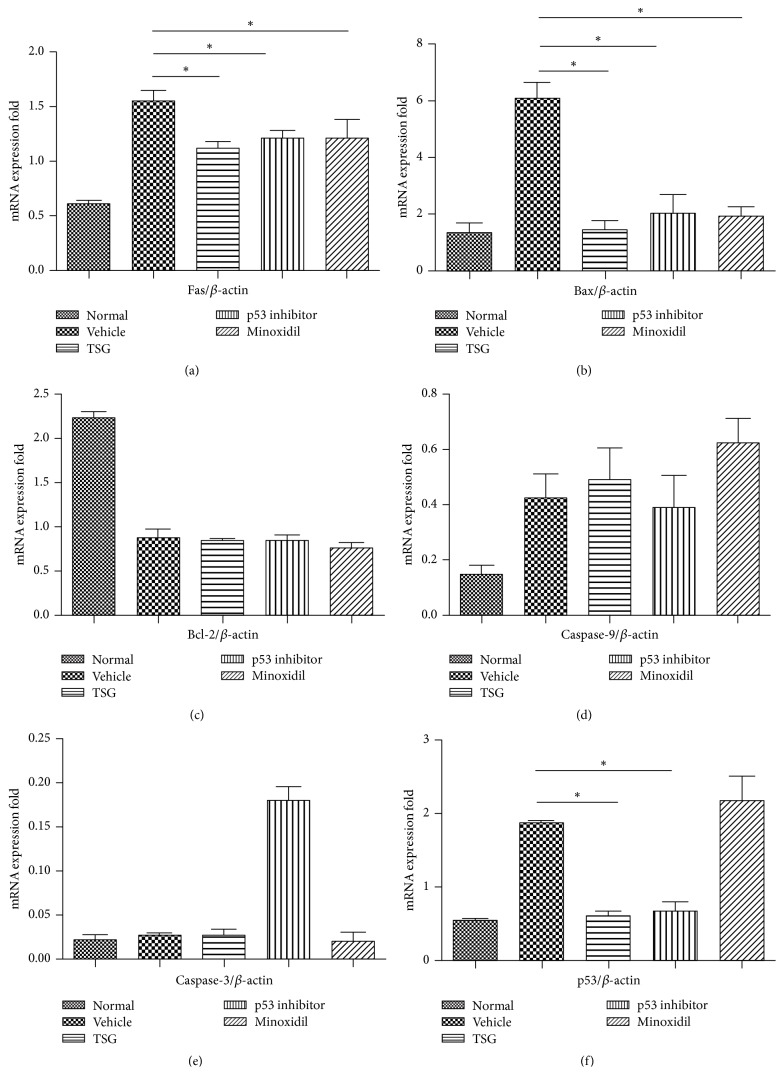
Expression of apoptosis related factors among five groups by quantitative real-time PCR analysis. Expression levels of Fas, Bax, and p53 were decreased after TSG treatment. ^*∗*^*P* < 0.01.

In both human (Figures 5(a) and 5(b)) and mice (Figures 6(a) and 6(b)) skin with hair loss, there was a notable increase in the expression levels of p53, Caspase-3, Active Caspase-3, and Caspase-9 and a significant decrease in the expression levels of anti-apoptotic factor of Bcl-2, which revealed that p53 and Bcl-2 family were involved in the hair loss. In order to identify which factors might contribute to the recovery of hair loss treated by TSG, apoptotic proteins were confirmed by western blot and the results exhibited that expression levels of p53 and Procaspase-9 were significantly decreased in TSG and p53 inhibitor groups as compared with vehicle group (Figures 6(a) and 6(b)). Additionally, only TSG could inhibit the expression of Active Caspase-3. Anti-apoptotic factor of Bcl-2 and proapoptotic factor of Bax are intrinsic pathway signaling molecules of apoptosis [5]. In mice treated with TSG, the protein expression of Bax was obviously decreased as compared with vehicle group (Figure 6(b)). Interestingly, there was no significant change of Bcl-2 between TSG and vehicle (Figures 6(a) and 6(b)). These results showed an increase of Bcl-2/Bax ratio in TSG group (Figure 6(b)) and implied that TSG could regulate Bax-mediated intrinsic apoptotic pathway when treating hair loss. Similar mRNA expression trends of p53 (Figure 7(f)) and Bax (Figure 7(b)) also appeared in TSG group using quantitative real-time PCR analysis. Furthermore, Fas gene was suppressed significantly in mice treated with TSG, p53 inhibitor, and Minoxidil (Figure 7(a)), which indicated that inhibition of Fas-mediated apoptotic pathway might also involve the hair regrowth in these three groups.

## 4. Discussion

Hair loss is the result of interaction of genetic, endocrine, and aging factors, which can be viewed as the abnormal control of catagen [20]. During catagen stage, hair follicles go through a highly controlled process of involution that largely reflects a burst of apoptosis [4, 21]. The molecular mechanisms of apoptosis are including extrinsic and intrinsic pathway. The extrinsic pathway is activated by binding of specific ligands such as Fas and TNF superfamily ligands [22, 23]. Our previous study has also found that Fas is significantly upregulated in human with alopecia areata through qPCR array [24]. The intrinsic pathway is controlled by Bcl-2 family, which also finally leads to the release of cytochrome c from mitochondria [25].

Fas-mediated extrinsic pathway occurs in the catagen stage. Fas L and Fas receptor recruit Fas-associated death domain (FADD) to induce Fas-FADD binding and subsequently activate the Caspase family, which is essential for apoptosis [22, 23]. In this study, we observed that TSG obviously ameliorated apoptosis through TUNEL staining. Through Western blot and Real-time RT-PCR analysis, we verified that TSG could not only inhibit Fas induced extrinsic apoptotic pathway but inhibit Bax induced intrinsic pathway to promote hair growth. Besides, p53 expression was also decreased after TSG treatment. P53 is a tumor suppressor protein that regulates the cell cycle and apoptosis [26]. The cross-talk between p53 and Fas pathways in modulating apoptosis has been suggested by several studies [27–29]. Thus, it is likely that TSG works through reducing p53 expression to inhibit Fas pathway to inhibit extrinsic apoptotic pathway.

In addition, we also noticed that local treatment with Pifithrin-*α* (acting as a p53 inhibitor) exhibited excellent stimulatory effects on hair restoration and growth, though less effective than TSG. We thus presumed that p53 inhibitor might also provide a new therapy for hair loss disorders.

Pharmacological studies have demonstrated that TSG have multiple biological functions in treating many diseases such as vascular and cardiac remodeling [30, 31], delaying the senescence effect [32], learning and memory disorders [33], and diabetic complication [34]. In this experiment, TSG showed great effects on hair growth. Treatment with TSG resulted in the emergence of new hair shafts and recoveries of the size and numbers of follicles. Few TUNEL^+^ cells were detected in the dorsal depilated skin treated with TSG. The next step of this experiment is going to find the dose-effect relationship of TSG in hair length and hair recovery ratio and figure out optimal drug concentration and delivery devices with the least side effects.

Taken together, these findings suggested that TSG could inhibit p53, Fas, and Bax induced apoptotic signaling pathway and help to prevent follicles from entering into catagen phase of the hair cycle. TSG may be an alternative medicine for treating hair loss such as alopecia and telogen effluvium.
